# Diaqua­bis­[2,6-bis­(4*H*-1,2,4-triazol-4-yl)pyridine-κ*N* ^2^]bis­(seleno­cyanato-κ*N*)cobalt(II)

**DOI:** 10.1107/S1600536812032461

**Published:** 2012-07-21

**Authors:** Yuan-Yuan Liu, Pan Yang

**Affiliations:** a Tianjin Key Laboratory of Structure and Performance for Functional Molecule, Tianjin Normal University, Tianjin 300071, People’s Republic of China

## Abstract

In the title compound, [Co(NCSe)_2_(C_9_H_7_N_7_)_2_(H_2_O)_2_], the Co^2+^ cation is coordinated by two seleno­cyanate anions, two 2,6-bis­(4*H*-1,2,4-triazol-4-yl)pyridine ligands and two water mol­ecules within a slightly distorted N_4_O_2_ octa­hedron. The asymmetric unit consists of one Co^2+^ cation, which is located on a center of inversion, as well as one seleno­cyanate anion, one 2,6-bis­(4*H*-1,2,4-triazol-4-yl)pyridine ligand and one water mol­ecule in general positions. Inter­molecular O—H⋯N hydrogen bonds join the complex mol­ecules into layers parallel to the *bc* plane. The layers are linked by C—H⋯N and C—H⋯Se hydrogen bonds into a three-dimensional supra­molecular architecture.

## Related literature

For general background to this work, see: Liu *et al.* (2007 ▶). Previous research on compounds with Co(II) as cation have found a slow relaxation of the magnetization, see: Boeckmann & Näther (2010, ▶ 2011 ▶, 2012) ▶. For related structures, see: Du *et al.* (2009 ▶); Yang *et al.* (2008 ▶).
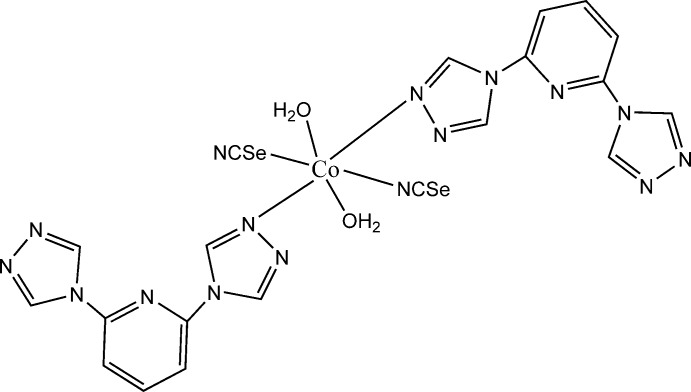


## Experimental

### 

#### Crystal data

[Co(NCSe)_2_(C_9_H_7_N_7_)_2_(H_2_O)_2_]
*M* *_r_* = 731.35
Monoclinic, 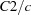

*a* = 17.5460 (16) Å
*b* = 7.2752 (7) Å
*c* = 20.3148 (19) Å
β = 95.691 (2)°
*V* = 2580.4 (4) Å^3^
*Z* = 4
Mo *K*α radiation
μ = 3.54 mm^−1^
*T* = 173 K
0.15 × 0.14 × 0.13 mm

#### Data collection

Bruker APEXII CCD diffractometer
Absorption correction: multi-scan (*SADABS*; Sheldrick, 1996 ▶) *T*_min_ = 0.619, *T*_max_ = 0.656
6336 measured reflections
2280 independent reflections
2083 reflections with *I* > 2σ(*I*)
*R*_int_ = 0.026

#### Refinement

*R*[*F* ^2^ > 2σ(*F* ^2^)] = 0.026
*wR*(*F* ^2^) = 0.065
*S* = 1.05
2280 reflections
186 parameters
H-atom parameters constrained
Δρ_max_ = 0.59 e Å^−3^
Δρ_min_ = −0.58 e Å^−3^

### 

Data collection: *APEX2* (Bruker, 2007 ▶); cell refinement: *SAINT* (Bruker, 2007 ▶); data reduction: *SAINT*; program(s) used to solve structure: *SHELXS97* (Sheldrick, 2008 ▶); program(s) used to refine structure: *SHELXL97* (Sheldrick, 2008 ▶); molecular graphics: *SHELXTL* and *DIAMOND* (Brandenburg, 1999 ▶); software used to prepare material for publication: *publCIF* (Westrip, 2010 ▶).

## Supplementary Material

Crystal structure: contains datablock(s) global, I. DOI: 10.1107/S1600536812032461/zj2088sup1.cif

Structure factors: contains datablock(s) I. DOI: 10.1107/S1600536812032461/zj2088Isup2.hkl

Additional supplementary materials:  crystallographic information; 3D view; checkCIF report

## Figures and Tables

**Table 1:** Selected bond lengths (Å)

Co1—N8	2.097 (2)
Co1—N3	2.122 (2)
Co1—O1	2.1434 (18)

**Table 2:** Hydrogen-bond geometry (Å, °)

*D*—H⋯*A*	*D*—H	H⋯*A*	*D*⋯*A*	*D*—H⋯*A*
O1—H1*A*⋯N2	0.84	2.43	3.017 (3)	127
O1—H1*B*⋯N7^ii^	0.84	2.00	2.837 (3)	173
C1—H1⋯N6^iii^	0.95	2.37	3.293 (3)	163
C5—H5⋯N8^iv^	0.95	2.56	3.356 (3)	142
C7—H7⋯N6^iii^	0.95	2.46	3.373 (3)	162
C9—H9⋯Se1^iv^	0.95	2.96	3.877 (3)	164

Symmetry codes: (ii) 
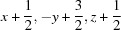
; (iii) 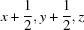; (iv) 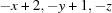.

**Fig. 1.**
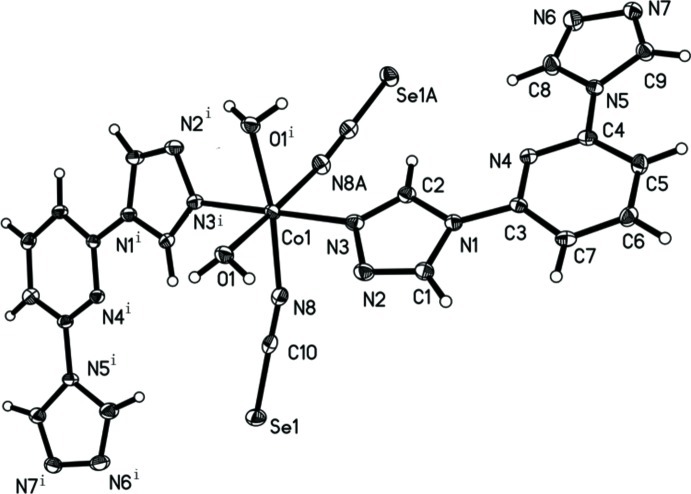
The structure of the title complex, showing 50% probability displacement ellipsoids and the atom-numbering schemes. Atoms of the inversion-related half-complex have symmetry code: (2 - x, y, 1/2 - z).

**Fig. 2.**
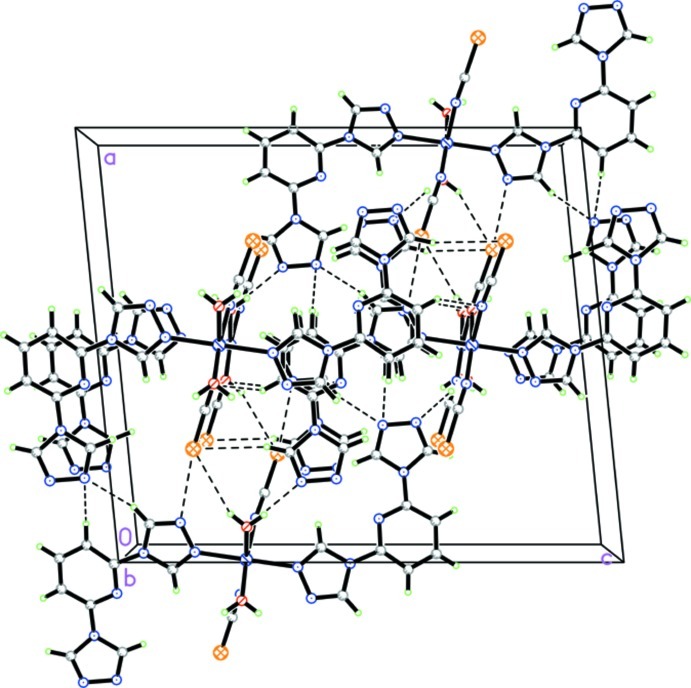
The three-dimensional layer structure of the title complex. Purple Dashed lines indicate O—H···N, C—H···N and C—H···Se hydrogen bonds.

## supplementary crystallographic information

### Comment

Previously we have reported on the a series of novel zinc(II) and cadmium(II) compounds based on 2,6-di-(1,2,4-triazole-4-yl)pyridine (Liu *et al.*, 2007). On the other hand, dependent on the nature of the metal cation, anti- or ferromagnetic ordering is observed and for the compounds with Co(II) as cation preivous resrarch have found a slow relaxation of the magnetization (Boeckmann & Näther 2010, 2011, 2012). To investigate the influence of the co-ligand on the magnetic properties for the compounds with Co(II), we tried to prepare cobalt(II) compounds based on 2,6-di-(1,2,4-triazole-4-yl)pyridine, which resulted in the formation of the title compound in which the neutral ligands are only terminal N-coordinated. This compound was characterized only by single-crystal X-ray diffraction. In the crystal structure the cobalt(II) cations are coordinated by four nitrogen atoms of two terminal N-bonded seleno-cyanato anions and two terminal bonded 2,6-di-(1,2,4-triazole-4-yl)pyridine co-ligands as well as two water molecules into discrete complexes (Fig. 1). The coordination polyhedron of the Co cations can be described as a slightly distorted octahedron with the Co cation located on a centre of inversion. The discrete cobalt complexes are bridged by intermolecular O—H···N, C—H···N and C—H···Se hydrogen bonds (Yang *et al.*, 2008; Du *et al.*, 2009), wich assemble (I) into a three-dimensional supra-molecular architecture(Fig. 2 and Table 1).

Perspective drawing with the atomic numbering scheme is illustrated in figure 1. Selected geometric parameters (Å, °) for (I) are listed in table 1. Selected hydrogen-bonding geometric parameters (Å, °) for (I) are listed in table 2. The two-dimensional supramolecular framework of (I) is shown in Figure 2.

### Experimental

The compound was synthesized under hydrothermal conditions. A mixture of *L* (*L* = 1,4-Bis(2,6-Bis(4*H*-1,2,4-triazol-4-yl)pyridine) (0.3 mmol, 0.0636 g), CoSO_4_.7H_2_O (0.1 mmol, 0.028 g), KSeCN (0.2 mmol, 0.029 g) and water (10 ml) was placed in a 25 ml acid digestion bomb and heated at 393 K for two days, then equably cooled to room temperature for three days. After washed by 5 ml water for twice, Red block crystals of the compound were obtained..

### Refinement

The water H atoms were located in a Fourier difference map and refined subject to an O—H restraint 0.88 (1) Å and an H···H restraint of 1.42 (2) Å. Other H atoms were allowed to ride on their parent atoms with C—H distances of 0.93 Å (*U*_iso_(H) = 1.2Ueq(C)). All of the non-hydrogen atoms were refined anisotropically..

### Crystal data

**Table:**

[Co(NCSe)_2_(C_9_H_7_N_7_)_2_(H_2_O)_2_]	*Z* = 4
*M**_r_* = 731.35	*F*(000) = 1444
Monoclinic, *C*2/*c*	*D*_x_ = 1.883 Mg m^−^^3^
Hall symbol: -C 2yc	Mo *K*α radiation, λ = 0.71073 Å
*a* = 17.5460 (16) Å	µ = 3.54 mm^−^^1^
*b* = 7.2752 (7) Å	*T* = 173 K
*c* = 20.3148 (19) Å	Block, red
β = 95.691 (2)°	0.15 × 0.14 × 0.13 mm
*V* = 2580.4 (4) Å^3^	

### Data collection

**Table:**

Bruker APEXII CCD diffractometer	2280 independent reflections
Radiation source: fine-focus sealed tube	2083 reflections with *I* > 2σ(*I*)
Graphite monochromator	*R*_int_ = 0.026
φ and ω scans	θ_max_ = 25.0°, θ_min_ = 2.3°
Absorption correction: multi-scan (*SADABS*; Sheldrick, 1996)	*h* = −20→9
*T*_min_ = 0.619, *T*_max_ = 0.656	*k* = −8→8
6336 measured reflections	*l* = −23→24

### Refinement

**Table:**

Refinement on *F*^2^	0 restraints
Least-squares matrix: full	H-atom parameters constrained
*R*[*F*^2^ > 2σ(*F*^2^)] = 0.026	*w* = 1/[σ^2^(*F*_o_^2^) + (0.0325*P*)^2^ + 4.1522*P*] where *P* = (*F*_o_^2^ + 2*F*_c_^2^)/3
*wR*(*F*^2^) = 0.065	(Δ/σ)_max_ = 0.002
*S* = 1.05	Δρ_max_ = 0.59 e Å^−^^3^
2280 reflections	Δρ_min_ = −0.58 e Å^−^^3^
186 parameters	

### Special details

**Table:**

Geometry. All e.s.d.'s (except the e.s.d. in the dihedral angle between two l.s. planes) are estimated using the full covariance matrix. The cell e.s.d.'s are taken into account individually in the estimation of e.s.d.'s in distances, angles and torsion angles; correlations between e.s.d.'s in cell parameters are only used when they are defined by crystal symmetry. An approximate (isotropic) treatment of cell e.s.d.'s is used for estimating e.s.d.'s involving l.s. planes.
Refinement. Refinement of *F*^2^ against ALL reflections. The weighted *R*-factor *wR* and goodness of fit *S* are based on *F*^2^, conventional *R*-factors *R* are based on *F*, with *F* set to zero for negative *F*^2^. The threshold expression of *F*^2^ > σ(*F*^2^) is used only for calculating *R*-factors(gt) *etc*. and is not relevant to the choice of reflections for refinement. *R*-factors based on *F*^2^ are statistically about twice as large as those based on *F*, and *R*- factors based on ALL data will be even larger.

### Fractional atomic coordinates and isotropic or equivalent isotropic displacement parameters (Å^2^)

**Table:**

	*x*	*y*	*z*	*U*_iso_*/*U*_eq_	
Co1	1.0000	0.85337 (6)	0.2500	0.01309 (13)	
Se1	1.238743 (15)	0.51140 (3)	0.329458 (13)	0.02053 (11)	
O1	1.08413 (10)	1.0675 (2)	0.26203 (8)	0.0171 (4)	
H1A	1.1136	1.0578	0.2321	0.026*	
H1B	1.1116	1.0623	0.2983	0.026*	
N1	1.01232 (11)	0.7890 (3)	0.04271 (10)	0.0134 (4)	
N2	1.08763 (12)	0.9006 (3)	0.12643 (10)	0.0171 (5)	
N3	1.01748 (11)	0.8524 (3)	0.14815 (10)	0.0143 (4)	
N4	0.91149 (11)	0.6660 (3)	−0.02481 (10)	0.0125 (4)	
N5	0.80409 (12)	0.5469 (3)	−0.08553 (10)	0.0136 (4)	
N6	0.69319 (13)	0.4701 (3)	−0.05234 (12)	0.0217 (5)	
N7	0.68675 (13)	0.4682 (3)	−0.12169 (11)	0.0182 (5)	
N8	1.09050 (12)	0.6689 (3)	0.27341 (10)	0.0180 (5)	
C1	1.08281 (14)	0.8611 (3)	0.06371 (12)	0.0156 (5)	
H1	1.1228	0.8797	0.0361	0.019*	
C2	0.97369 (14)	0.7874 (3)	0.09813 (12)	0.0139 (5)	
H2	0.9227	0.7452	0.0998	0.017*	
C3	0.98360 (13)	0.7256 (3)	−0.02156 (12)	0.0114 (5)	
C4	0.88145 (14)	0.6091 (3)	−0.08405 (12)	0.0135 (5)	
C5	0.91929 (15)	0.6072 (3)	−0.14054 (12)	0.0160 (5)	
H5	0.8949	0.5656	−0.1817	0.019*	
C6	0.99442 (14)	0.6689 (3)	−0.13447 (12)	0.0163 (5)	
H6	1.0226	0.6702	−0.1720	0.020*	
C7	1.02849 (14)	0.7287 (3)	−0.07389 (12)	0.0150 (5)	
H7	1.0801	0.7698	−0.0685	0.018*	
C8	0.76306 (16)	0.5177 (3)	−0.03236 (14)	0.0199 (6)	
H8	0.7829	0.5305	0.0126	0.024*	
C9	0.75337 (15)	0.5140 (3)	−0.13963 (13)	0.0171 (6)	
H9	0.7651	0.5233	−0.1842	0.020*	
C10	1.14850 (15)	0.6077 (3)	0.29588 (12)	0.0167 (5)	

### Atomic displacement parameters (Å^2^)

**Table:**

	*U*^11^	*U*^22^	*U*^33^	*U*^12^	*U*^13^	*U*^23^
Co1	0.0104 (2)	0.0163 (2)	0.0123 (2)	0.000	−0.00015 (19)	0.000
Se1	0.01776 (16)	0.02176 (16)	0.02165 (17)	0.00585 (11)	−0.00019 (12)	0.00176 (10)
O1	0.0126 (8)	0.0238 (9)	0.0150 (9)	−0.0010 (8)	0.0014 (7)	−0.0007 (7)
N1	0.0111 (10)	0.0154 (10)	0.0138 (11)	−0.0007 (8)	0.0017 (8)	−0.0003 (8)
N2	0.0118 (11)	0.0201 (10)	0.0192 (12)	−0.0021 (9)	0.0015 (9)	−0.0001 (9)
N3	0.0113 (10)	0.0159 (10)	0.0158 (11)	−0.0006 (8)	0.0020 (9)	0.0017 (8)
N4	0.0110 (10)	0.0127 (9)	0.0138 (10)	0.0004 (8)	0.0017 (8)	0.0005 (8)
N5	0.0101 (10)	0.0156 (10)	0.0150 (11)	−0.0016 (8)	0.0012 (9)	−0.0010 (8)
N6	0.0156 (11)	0.0293 (12)	0.0205 (12)	−0.0058 (10)	0.0033 (10)	−0.0008 (10)
N7	0.0151 (11)	0.0208 (11)	0.0185 (12)	−0.0006 (9)	0.0005 (9)	−0.0004 (9)
N8	0.0191 (12)	0.0200 (10)	0.0151 (11)	0.0031 (10)	0.0023 (9)	−0.0022 (9)
C1	0.0121 (12)	0.0177 (12)	0.0171 (13)	−0.0028 (10)	0.0017 (10)	−0.0015 (10)
C2	0.0122 (11)	0.0159 (12)	0.0140 (12)	−0.0007 (10)	0.0032 (10)	0.0016 (10)
C3	0.0105 (11)	0.0089 (11)	0.0145 (12)	0.0003 (9)	−0.0002 (10)	0.0012 (9)
C4	0.0106 (12)	0.0108 (11)	0.0189 (13)	−0.0008 (10)	0.0007 (10)	0.0007 (10)
C5	0.0182 (13)	0.0155 (11)	0.0145 (12)	−0.0021 (10)	0.0021 (10)	−0.0032 (10)
C6	0.0174 (13)	0.0181 (12)	0.0140 (12)	0.0005 (10)	0.0055 (10)	0.0013 (10)
C7	0.0104 (11)	0.0157 (12)	0.0190 (13)	0.0000 (10)	0.0021 (10)	0.0022 (10)
C8	0.0171 (14)	0.0266 (14)	0.0161 (14)	−0.0047 (11)	0.0019 (11)	−0.0026 (10)
C9	0.0157 (13)	0.0193 (13)	0.0160 (14)	−0.0017 (10)	0.0002 (11)	−0.0005 (10)
C10	0.0198 (14)	0.0157 (12)	0.0152 (13)	0.0000 (11)	0.0052 (11)	−0.0036 (10)

### Geometric parameters (Å, º)

**Table:**

Co1—N8^i^	2.097 (2)	N5—C8	1.373 (3)
Co1—N8	2.097 (2)	N5—C4	1.428 (3)
Co1—N3^i^	2.122 (2)	N6—C8	1.300 (4)
Co1—N3	2.122 (2)	N6—N7	1.402 (3)
Co1—O1	2.1434 (18)	N7—C9	1.302 (3)
Co1—O1^i^	2.1433 (17)	N8—C10	1.162 (3)
Se1—C10	1.803 (3)	C1—H1	0.9500
O1—H1A	0.8400	C2—H2	0.9500
O1—H1B	0.8400	C3—C7	1.385 (3)
N1—C2	1.371 (3)	C4—C5	1.382 (3)
N1—C1	1.372 (3)	C5—C6	1.387 (4)
N1—C3	1.429 (3)	C5—H5	0.9500
N2—C1	1.301 (3)	C6—C7	1.384 (3)
N2—N3	1.393 (3)	C6—H6	0.9500
N3—C2	1.300 (3)	C7—H7	0.9500
N4—C4	1.331 (3)	C8—H8	0.9500
N4—C3	1.333 (3)	C9—H9	0.9500
N5—C9	1.365 (3)		
			
N8^i^—Co1—N8	100.44 (12)	C9—N7—N6	107.1 (2)
N8^i^—Co1—N3^i^	92.26 (8)	C10—N8—Co1	161.5 (2)
N8—Co1—N3^i^	87.48 (8)	N2—C1—N1	111.0 (2)
N8^i^—Co1—N3	87.48 (8)	N2—C1—H1	124.5
N8—Co1—N3	92.26 (8)	N1—C1—H1	124.5
N3^i^—Co1—N3	179.60 (11)	N3—C2—N1	109.7 (2)
N8^i^—Co1—O1	171.22 (7)	N3—C2—H2	125.2
N8—Co1—O1	86.66 (7)	N1—C2—H2	125.2
N3^i^—Co1—O1	93.20 (7)	N4—C3—C7	125.2 (2)
N3—Co1—O1	87.09 (7)	N4—C3—N1	113.4 (2)
N8^i^—Co1—O1^i^	86.66 (7)	C7—C3—N1	121.4 (2)
N8—Co1—O1^i^	171.22 (7)	N4—C4—C5	125.0 (2)
N3^i^—Co1—O1^i^	87.09 (7)	N4—C4—N5	114.1 (2)
N3—Co1—O1^i^	93.20 (7)	C5—C4—N5	120.9 (2)
O1—Co1—O1^i^	86.76 (9)	C4—C5—C6	117.0 (2)
Co1—O1—H1A	108.8	C4—C5—H5	121.5
Co1—O1—H1B	113.3	C6—C5—H5	121.5
H1A—O1—H1B	106.9	C7—C6—C5	120.2 (2)
C2—N1—C1	104.6 (2)	C7—C6—H6	119.9
C2—N1—C3	126.1 (2)	C5—C6—H6	119.9
C1—N1—C3	129.3 (2)	C6—C7—C3	116.7 (2)
C1—N2—N3	106.3 (2)	C6—C7—H7	121.6
C2—N3—N2	108.43 (19)	C3—C7—H7	121.6
C2—N3—Co1	129.19 (17)	N6—C8—N5	110.3 (2)
N2—N3—Co1	121.72 (15)	N6—C8—H8	124.8
C4—N4—C3	115.8 (2)	N5—C8—H8	124.8
C9—N5—C8	104.8 (2)	N7—C9—N5	110.6 (2)
C9—N5—C4	127.9 (2)	N7—C9—H9	124.7
C8—N5—C4	127.2 (2)	N5—C9—H9	124.7
C8—N6—N7	107.2 (2)	N8—C10—Se1	179.1 (2)
			
C1—N2—N3—C2	−0.3 (3)	C4—N4—C3—C7	−1.3 (3)
C1—N2—N3—Co1	171.20 (16)	C4—N4—C3—N1	178.59 (19)
N8^i^—Co1—N3—C2	12.0 (2)	C2—N1—C3—N4	2.0 (3)
N8—Co1—N3—C2	112.4 (2)	C1—N1—C3—N4	−179.1 (2)
N3^i^—Co1—N3—C2	62.2 (3)	C2—N1—C3—C7	−178.1 (2)
O1—Co1—N3—C2	−161.1 (2)	C1—N1—C3—C7	0.8 (4)
O1^i^—Co1—N3—C2	−74.5 (2)	C3—N4—C4—C5	0.3 (3)
N8^i^—Co1—N3—N2	−157.53 (17)	C3—N4—C4—N5	−179.81 (19)
N8—Co1—N3—N2	−57.18 (17)	C9—N5—C4—N4	166.4 (2)
N3^i^—Co1—N3—N2	−107.3 (3)	C8—N5—C4—N4	−9.4 (3)
O1—Co1—N3—N2	29.36 (17)	C9—N5—C4—C5	−13.7 (4)
O1^i^—Co1—N3—N2	115.95 (17)	C8—N5—C4—C5	170.5 (2)
C8—N6—N7—C9	0.3 (3)	N4—C4—C5—C6	0.3 (4)
N8^i^—Co1—N8—C10	−156.3 (7)	N5—C4—C5—C6	−179.6 (2)
N3^i^—Co1—N8—C10	−64.4 (6)	C4—C5—C6—C7	0.1 (4)
N3—Co1—N8—C10	115.9 (6)	C5—C6—C7—C3	−0.9 (3)
O1—Co1—N8—C10	28.9 (6)	N4—C3—C7—C6	1.6 (4)
O1^i^—Co1—N8—C10	−12.6 (10)	N1—C3—C7—C6	−178.2 (2)
N3—N2—C1—N1	0.2 (3)	N7—N6—C8—N5	−0.3 (3)
C2—N1—C1—N2	0.0 (3)	C9—N5—C8—N6	0.3 (3)
C3—N1—C1—N2	−179.1 (2)	C4—N5—C8—N6	176.8 (2)
N2—N3—C2—N1	0.3 (3)	N6—N7—C9—N5	−0.1 (3)
Co1—N3—C2—N1	−170.33 (15)	C8—N5—C9—N7	−0.1 (3)
C1—N1—C2—N3	−0.2 (3)	C4—N5—C9—N7	−176.6 (2)
C3—N1—C2—N3	178.9 (2)	Co1—N8—C10—Se1	−132 (15)

Symmetry code: (i) −*x*+2, *y*, −*z*+1/2.

### Hydrogen-bond geometry (Å, º)

**Table:**

*D*—H···*A*	*D*—H	H···*A*	*D*···*A*	*D*—H···*A*
O1—H1*A*···N2	0.84	2.43	3.017 (3)	127
O1—H1*B*···N7^ii^	0.84	2.00	2.837 (3)	173
C1—H1···N6^iii^	0.95	2.37	3.293 (3)	163
C5—H5···N8^iv^	0.95	2.56	3.356 (3)	142
C7—H7···N6^iii^	0.95	2.46	3.373 (3)	162
C9—H9···Se1^iv^	0.95	2.96	3.877 (3)	164

Symmetry codes: (ii) *x*+1/2, −*y*+3/2, *z*+1/2; (iii) *x*+1/2, *y*+1/2, *z*; (iv) −*x*+2, −*y*+1, −*z*.

## References

[bb1] Boeckmann, J. & Näther, C. (2010). *Dalton Trans.* **39**, 11019–11026. 10.1039/c0dt00904k 20949144

[bb2] Boeckmann, J. & Näther, C. (2011). *Chem. Commun.* **47**, 7104–7106. 10.1039/c1cc12273h 21617809

[bb3] Boeckmann, J. & Näther, C. (2012). *Polyhedron*, **31**, 587–595.

[bb4] Brandenburg, K. (1999). *DIAMOND* Crystal Impact GbR, Bonn, Germany.

[bb5] Bruker (2007). *APEX2* and *SAINT* Bruker AXS Inc., Madison, Wisconsin, USA.

[bb6] Du, Z.-Y., Sun, Y.-H., Liu, Q.-Y., Xie, Y.-R. & &Wen, H.-R. (2009). *Inorg. Chem.* **48**, 7015–7017. 10.1021/ic901130a 19583245

[bb7] Liu, Y. Y., Huang, Y. Q., Shi, W., Cheng, P., Liao, D. Z.& Yan, S. P. (2007). *Cryst. Growth Des.* **7**, 1483–1489.

[bb8] Sheldrick, G. M. (1996). *SADABS* University of Göttingen, Germany.

[bb9] Sheldrick, G. M. (2008). *Acta Cryst.* A**64**, 112–122. 10.1107/S0108767307043930 18156677

[bb10] Westrip, S. P. (2010). *J. Appl. Cryst.* **43**, 920–925.

[bb11] Yang, B.-P., Prosvirin, A. V., Guo, Y.-Q. & Mao, J.-G. (2008). *Inorg. Chem.* **47**, 1453–1459. 10.1021/ic701351x 18225859

